# An innovative magnetic state generator using machine learning techniques

**DOI:** 10.1038/s41598-019-53411-y

**Published:** 2019-11-13

**Authors:** H. Y. Kwon, N. J. Kim, C. K. Lee, H. G. Yoon, J. W. Choi, C. Won

**Affiliations:** 1 Department of Physics, Kyung Hee University, Seoul, 02447 South Korea; 2 Center for Spintronics, Korea Institute of Science and Technology, Seoul, 02792 South Korea

**Keywords:** Ferromagnetism, Information theory and computation

## Abstract

We propose a new efficient algorithm to simulate magnetic structures numerically. It contains a generative model using a complex-valued neural network to generate *k*-space information. The output information is hermitized and transformed into *real*-space spin configurations through an inverse fast Fourier transform. The Adam version of stochastic gradient descent is used to minimize the magnetic energy, which is the cost of our algorithm. The algorithm provides the proper ground spin configurations with outstanding performance. In model cases, the algorithm was successfully applied to solve the spin configurations of magnetic chiral structures. The results also showed that a magnetic long-range order could be obtained regardless of the total simulation system size.

## Introduction

Recently, machine learning (ML), a computational technique used to give computer systems the ability to learn, has been applied to numerous research disciplines, including physics. Magnetism, in particular, is one of the main research fields of condensed matter physics, and various ML techniques have been applied to study fundamental topics in magnetism, such as the phase transition of a spin system^1–4^, quantum magnetic states^5,6^, and classical spin system for magnetic domains^7^.

Conventionally, magnetic structures are calculated by solving the magnetic Hamiltonian with the help of tools such as Monte-Carlo simulations, greedy algorithm, and spin dynamics simulations based on the Landau-Lifshitz-Gilbert equation to investigate the characteristics of various magnetic structures^8,9^. However, obtaining well-ordered magnetic states^10,11^ using these methods is not guaranteed because the solutions of those methods are only some of the multiple local stable states the magnetic systems can have. In particular, the difficulty of obtaining a ground state increases when the systems contain various spin-spin interactions, axial properties, and directional biases; thus, they have infinitely numerous local stable states.

A recent study^7^ showed that ML techniques can be an efficient way to search for the magnetic ground state. The algorithm proposed in that study contains a simple generative ML model which can generate spin configurations, and the Adam version of stochastic gradient descent^12^ is performed to minimize the magnetic energy. This algorithm, performed in *real* space (*r*-space), showed state-of-the-art performance compared with conventional methods.

The concept of a complex-valued neural network (CVNN) was first proposed to improve the performance of ML algorithms through expanded degrees of freedom of network parameters^13–16^. A CVNN can also be used to solve physical systems expressed in the form of complex numbers, such as quantum many-body systems^5^.

The magnetic states can also be represented in the form of complex numbers using the *reciprocal* space (*k*-space) expression. This expression has an important advantage in magnetism research because various magnetic domain structures, such as the magnetic stripe domain^17^ and skyrmion lattice^10^, are observed with translational symmetries with specific length scales that are determined by the magnetic parameters of the system.

Therefore, in this paper, we propose a new spin configuration simulation algorithm utilizing the advantages of *k*-space. Our algorithm, a *k*-space-based algorithm, generates complex values in *k*-space through a simple CVNN. The generated *k*-space values are hermitized and transformed into *r*-space values through an inverse fast Fourier transform (IFFT). The *r*-space values are regarded as components of Heisenberg spins, and the magnetic Hamiltonian is applied to calculate the magnetic energy. We performed the Adam version of stochastic gradient descent to minimize the magnetic energy. Generating classical Heisenberg spin configurations using an ML algorithm composed of a CVNN is an unprecedented approach in the magnetism research field.

Compared with an *r*-space-based algorithm^7^ that showed the previous state-of-the-art performance, our *k*-space-based algorithm generates magnetic ground states more efficiently. The results also show long-range ordered structures regardless of the total simulation system size.

## Simulation Model

Our goal is to obtain ground state magnetic structures using a magnetic Hamiltonian that generally contains exchange interaction, Dzyaloshinskii-Moriya interaction (DMI)^18,19^, dipolar interaction, anisotropies, and Zeeman energy. Before considering the general case, for the quantitative investigation of the characteristics of the *k*-space-based algorithm, we first applied it to a simple magnetic system where the analytical solutions were known by previous studies^20,21^. The Hamiltonian, ${ {\mathcal H} }_{J,DM}$, used to describe the simple system contains only the exchange interaction and DMI, and is expressed as:

1 $${ {\mathcal H} }_{J,DM}=-\,J\sum _{\langle i,j\rangle }{\overrightarrow{S}}_{i}\cdot {\overrightarrow{S}}_{j}-\sum _{\langle i,j\rangle }{\overrightarrow{DM}}_{ij}\cdot ({\overrightarrow{S}}_{i}\times {\overrightarrow{S}}_{j})$$

where *J* and ${\overrightarrow{DM}}_{ij}$ are the strength of the exchange interaction and the Dzyaloshinskii-Moriya vectors, respectively. *J* and $|{\overrightarrow{DM}}_{ij}|$ are written in units of energy. We used the classical Heisenberg spin model, which is one of the general spin models for studying magnetic domains and magnetic properties^20–25^. In the Heisenberg model, the spin $\overrightarrow{S}$ is considered a three-dimensional unit vector. When the energy minimization conditions are calculated analytically according to Eq. (1), it is known that the ground states of the two-dimensional magnetic systems are sinusoidal wave profiles, such as helical or cycloidal structures^20,21^.

After verifying our algorithm with known analytical solutions, as the next step, we generalized the *k*-space-based algorithm to obtain the magnetic configurations formed in a more complicated system, such as a quasi-two-dimensional system, in which the total Hamiltonian is expressed as:

2 $$\begin{array}{rcl} {\mathcal H}  & = & { {\mathcal H} }_{J,DM}-{K}_{{\rm{u}}}\sum _{i}{|{\overrightarrow{S}}_{i,{\rm{u}}}|}^{2}-{\overrightarrow{h}}_{{\rm{ext}}}\cdot \sum _{i}{\overrightarrow{S}}_{i}\\  &  & -\,{D}_{{\rm{dip}}}\sum _{i\ne j}\frac{3({\overrightarrow{S}}_{i}\cdot {\overrightarrow{r}}_{ij})({\overrightarrow{S}}_{j}\cdot {\overrightarrow{r}}_{ij})-({\overrightarrow{S}}_{i}\cdot {\overrightarrow{S}}_{j}){|{\overrightarrow{r}}_{ij}|}^{2}}{{|{\overrightarrow{r}}_{ij}|}^{5}}\end{array}$$

where *K*_u_ and *D*_dip_ are the strength of the uniaxial anisotropy and dipolar interaction, and ${\overrightarrow{h}}_{{\rm{ext}}}$ is the applied external magnetic field. We used a square or cubic grid model to simulate two- or three-dimensional magnetic systems, so ${\overrightarrow{r}}_{ij}$ is a dimensionless displacement vector between *i* and *j* grid sites.

## Machine Learning Algorithm

**Figure 1:**
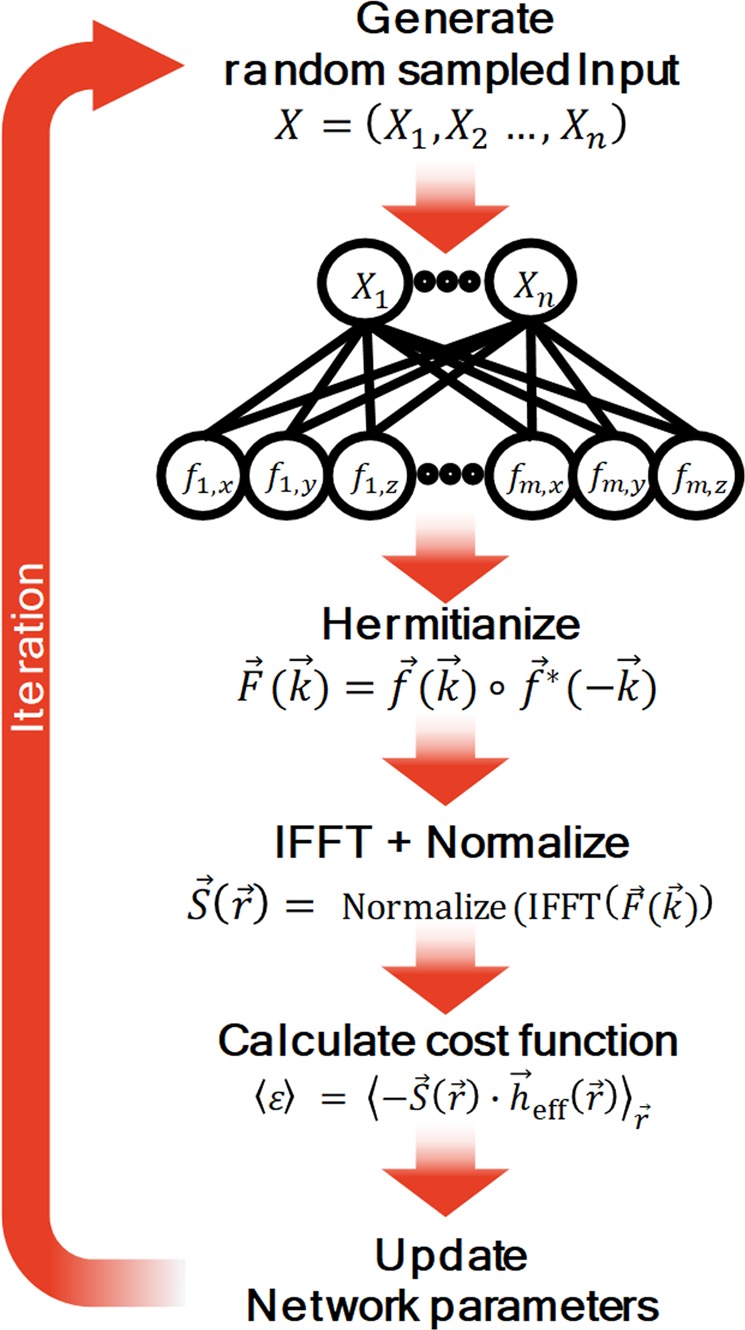
Iterative processes in the k-space-based algorithm to obtain energy minimized spin configurations using ML techniques.

ML algorithm was used to generate *k*-space information of the spin configurations, and the total iteration process is shown in Fig. 1. The input *X* in Fig. 1 is composed of *n* randomly sampled values from the standard normal distribution for the stochastic behavior of the optimization process.

A simple complex-valued one-layer perceptron, which is the simplest CVNN, is used to obtain output *k*-space information *f*. The number of *f* values is *m* × 3, where *m* is the total number of grid sites in *k*-space. In other words, *n* and *m* × 3 are the numbers of input and output data for the one-layer CVNN, respectively. We used a one-layer CVNN rather than a multilayered CVNN because of two reasons. One is that one layered neural network structure is enough to generate energy minimized spin configurations as shown in the previous study^7^. The simple network structure of that study has the advantage of being able to investigate in detail the changes in the network parameters that occurred during the training process. The other is that the effect of the multilayer in our algorithm did not improve the results. The spin configurations from the multilayered CVNN were frozen in local energy minimum states, which means that the stochastic behavior of the algorithm from the input random numbers in *X* was reduced due to the additional layers.

We set the components of the network parameters, weight and bias, as complex numbers to represent the phases of *k*-space information; thus, each of the *f* values is composed of complex numbers. This information is reshaped to a three-dimensional *k*-space vector configuration $\overrightarrow{F}(\overrightarrow{k})$ with $[{{\rm{L}}}_{x},\,{{\rm{L}}}_{y},\,{{\rm{L}}}_{z},\,3]$ dimensionality using the *k*-space lattice vector $\overrightarrow{k}={a}_{1}\hat{i}+{a}_{2}\hat{j}+{a}_{3}\hat{k}$, where *a*_1_, *a*_2_, $\,{a}_{3}\in {\mathbb{Z}}$. The $\hat{i}$, $\,\hat{j}$, and $\hat{k}$ are the unit vectors of the $x,\,y$ and $z$ axes of *k*-space respectively.

After the reshaping process for $\overrightarrow{f}(\overrightarrow{k})$, a Hermitian function, $\overrightarrow{F}(\overrightarrow{k})$, is calculated using the hermitization method $\overrightarrow{F}(\overrightarrow{k})=\overrightarrow{f}(\overrightarrow{k})\circ {\overrightarrow{f}}^{\ast }(\,-\,\overrightarrow{k})$, where $\circ$ represents the Hadamard product. There are two main reasons for using hermitization in the *k*-space-based algorithm. First, this method is used to obtain real number information of spin vectors in *r*-space. In the Heisenberg model, a spin is a three-dimensional vector with three real numbers. Hermitization results in an IFFT consisting of only real parts, so the IFFT results can be treated as *r*-space spin vectors in the Heisenberg model. Second, hermitization adds a nonlinear operation defined in complex number space. The operation can emphasize differences in the *k*-space values, and it is expected to be advantageous in searching for the periodicity of the *r*-space spin configurations. The novelties of this study are the use of a one-layer CVNN and the use of hermitization operation to improve the performance of ML algorithm for generating magnetic spin configurations. The *r*-space spin configuration $\overrightarrow{S}(\overrightarrow{r})$ is calculated from the IFFT of $\overrightarrow{F}(\overrightarrow{k})$. The magnetic Hamiltonian is used to obtain the energy density value $\langle \varepsilon \rangle ={\langle -\overrightarrow{S}(\overrightarrow{r})\cdot {\overrightarrow{h}}_{{\rm{eff}}}(\overrightarrow{r})\rangle }_{\overrightarrow{r}}$ of the system.

We set the total cost, which should be minimized, to be $\langle \varepsilon \rangle +\frac{1}{2}\lambda W{W}^{T}$, where $\lambda \,(\,=\,{10}^{-4})$ is the regularization coefficient and *W* is a tensor for weight network parameter. Since the last term of the cost, $\frac{1}{2}\lambda W{W}^{T}$, can prevent the divergence of the magnitude of the network parameter through the cost minimization process, it can make the stochastically fluctuating behavior of our algorithm reduced gradually as training progresses. Hence, it is expected that the final spin configuration generated by our algorithm at the last iteration becomes a single stable spin configuration. We added an Adam optimizer at the last part of the *k*-space-based algorithm to minimize the cost, and the total process iterated 100,000 times with a 0.001 learning rate.

## Results and Discussion

**Figure 2:**
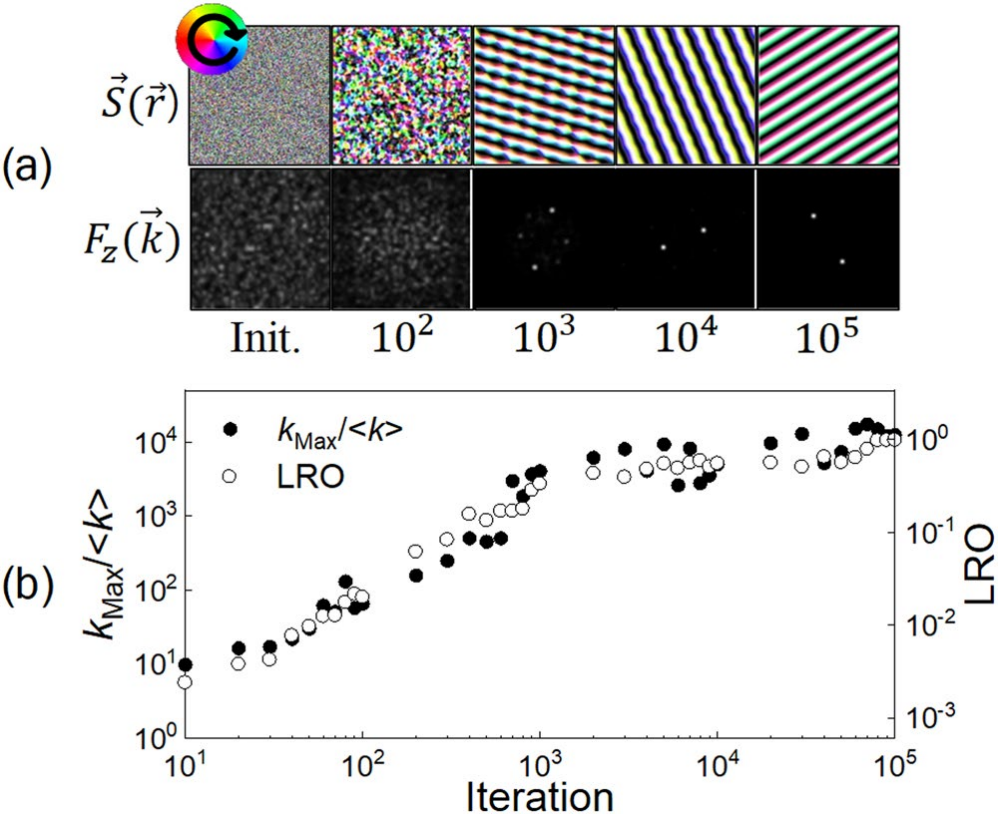
(**a**) The generated spin configurations at 10^0^(Init.), 10^2^, 10^3^, 10^4^ and 10^5^ iterations. The color wheel indicates in-plane magnetization directions, and white-black is for the out-of-plane spin directions. (**b**) The parameters showing the degree of long-range ordering of generated spin configurations. *k*_Max_ and $\langle k\rangle$ are the maximum and the average of *k*-space values for each iteration, respectively.

To investigate how the learning process of the *k*-space-based algorithm progresses quantitatively, we applied this algorithm to the simple system described by the Hamiltonian in Eq. (1) with *J* = 1, $|{\overrightarrow{DM}}_{ij}|=0.3$, $n=8$, and $m=4\times {10}^{4}$ (${{\rm{L}}}_{x}\times {{\rm{L}}}_{y}\times {{\rm{L}}}_{z}=200\times 200\times 1$) simulation conditions. The results are shown in Fig. 2.

Perfectly aligned chiral structures were obtained in 10^5^ iterations. At the early stage, small magnetic domain structures are formed from the initial random spin configuration. They are connected to become perfectly aligned chiral structures as the simulation progresses. The energy density of the spin configuration generated after 10^5^ iterations was calculated as $\langle \varepsilon \rangle \approx -\,2.044$, and the periodicity of this chiral structure, *l*, was approximately 21 grid sites. These values are very close to the ground state characteristics known from analytical solutions^20,21^, $\langle \varepsilon \rangle =-\,J-\sqrt{{J}^{2}+{|\overrightarrow{DM}|}^{2}}\cong -\,2.044$ and $l=2\pi /{\cos }^{-1}(\frac{J}{\sqrt{{J}^{2}+{|\overrightarrow{DM}|}^{2}}})\cong 21.56$ grid sites. The *z* component of *k*-space information, ${F}_{z}(\overrightarrow{k})$, exhibits the characteristics of the *r*-space spin configuration more clearly. ${F}_{z}(\overrightarrow{k})$ of the simulation was distributed randomly at the early stage and evolved to a feature with two bright peaks, which indicate a sinusoidal structure in *r*-space.

In addition to generating the magnetic structures with the morphological characteristics of the ground state, increasing the degree of ordering of the generated magnetic structures is also an important factor to minimize the system energy as experimentally observed well-ordered magnetic states^10,11^. We investigated two parameters to quantitatively analyze the ordering of the generated spin configurations. One is the magnitude of the peaks appearing in *k*-space, and the other is the long-range order (LRO) parameter^7,22^, which was calculated from the time correlation between spins at the current iteration and spins at the final state. Both values increased as the generated spin configuration became aligned Fig. 2(b). The magnetic structure was almost ordered in the initial ${10}^{3}$ iterations, which is a hundredth of the total number of iterations. This indicates that this algorithm utilizing *k*-space is very efficient in building long-range order at an early stage. As a comparison, when the ML technique was applied to directly generate the *r*-space spin configuration^7^, the LRO parameter was typically built up around 10^4^∼10^5^ iterations. Therefore, adopting a series of processes in *k*-space greatly improved the ML technique to generate long-range ordered spin configurations.

This algorithm was also found to be efficient in escaping from local minimum states. Notice that the period and the alignment direction of the spin configuration were not fixed during the simulation process, which implies that this algorithm can easily overcome the energy barrier needed to change the periodicity of magnetic structures. This property is important in studying the ground states of systems with hysteresis. The spin configuration is not inherited from the previous iteration since the algorithm basically converts the random input into the spin configuration through a trained neural network. Therefore, our algorithm has stochastic behavior that generates a different magnetic structure for every iteration to find a ground state without being affected by hysteresis characteristics.

Another advantage of the *k*-space-based algorithm is manifested in the large-scale simulations possible in *k*-space. As the system size of the simulations increases, unwanted boundary or size effects diminish, and the accuracy of the results significantly improves. A large-scale simulation is also required to investigate systems where magnetic structures with various length scales coexist. However, increasing the size of the simulations based on *r*-space can cause problems since the results are made up of domains built with local energy minimization, and the long-range order of the system is lost. We can overcome these problems by exploring magnetic structures in *k*-space, which can represent the characteristics of spin configurations consistently for various system sizes.

**Figure 3:**
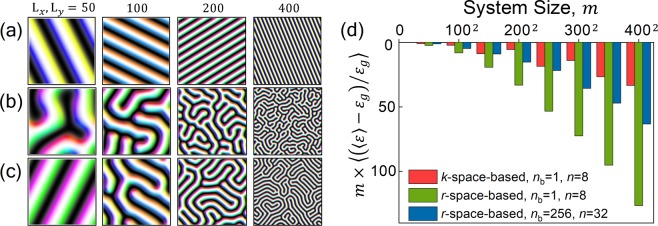
Generated spin configurations for the simulation systems with various lateral sizes L_*x*_ = L_*y*_ = 50, 100, 200, and 400 after 10^5^ iterations using (**a**) *k*-space-based algorithm (our algorithm) with *n* = 8, batch size *n*_*b*_ = 8, (**b**) *r*-space-based algorithm with *n* = 8, *n*_*b*_ = 1, and (**c**) *r*-space-based algorithm with *n* = 32, *n*_*b*_ = 256. (**d**) The differences among the analytic solutions of ground state energy, ${\varepsilon }_{{\rm{g}}}$, and simulated energy, $\langle \varepsilon \rangle$, using the three algorithms for each lateral size.

To verify this approach, we compared the results obtained with *k*-space-based and *r*-space-based algorithms for systems with various lateral sizes from ${{\rm{L}}}_{x}={{\rm{L}}}_{y}=50$ to 400 Fig. 3. Our *k*-space-based algorithm can perfectly simulate the aligned chiral structures regardless of system size Fig. 3(a). On the other hand, the spin configurations generated by the *r*-space-based algorithm with the same simulation conditions (batch size *n*_*b*_ = 1 and *n* = 1) showed less ordered structures Fig. 3(b). It is known that increasing the batch size and the size of random input is effective in generating ground states in an *r*-space-based algorithm^7^. We ran another simulation using the algorithm with batch sizes *n*_*b*_ = 256 and *n* = 32. The alignment of generated spin configurations improved Fig. 3(c), but the perfectly aligned ground state appearing in our *k*-space-based results Fig. 3(a) could still not be obtained. Moreover, increasing the batch size can cause a disadvantage of a longer iteration run time due to a large amount of the computational process. In our case, the computation run time for obtaining results using the *r*-space-based algorithm with the *n*_*b*_ = 256 condition is increased by 10 times compared to the case of *n*_*b*_ = 1 condition.

To clearly show the differences between the algorithms discussed in Fig. 3(a–c), the simulated energy from each of the algorithms is compared with the analytic solution Fig. 3(d). The results from the *k*-space-based algorithm are closer to the analytically calculated ground state energy than the results from both *r*-space-based algorithms for all system sizes. These results clearly show the correctness of the proposed algorithm in this study.

To show that the *k*-space-based algorithm can be used to investigate the ground state of a more complicated system, we applied the algorithm to obtain the ground state of the magnetic bubble domain formed in a quasi-two-dimensional system where the thickness can affect the magnetic domain profile. Since the magnetic bubble structures are coupled to each other by long-range dipolar interaction, the binding strength sharply decreases as the distance between the bubbles increases. Hence, it is difficult to obtain a perfectly aligned bubble domain with a certain periodicity and lattice structure through a numerical simulation method. In addition, the Neel cap regions, which are generated from the boundary effect of the dipolar interaction, can appear in a quasi-two-dimensional system^26–29^. Therefore, it is a suitable and challenging system that can be used to prove the performance of this algorithm.

**Figure 4:**
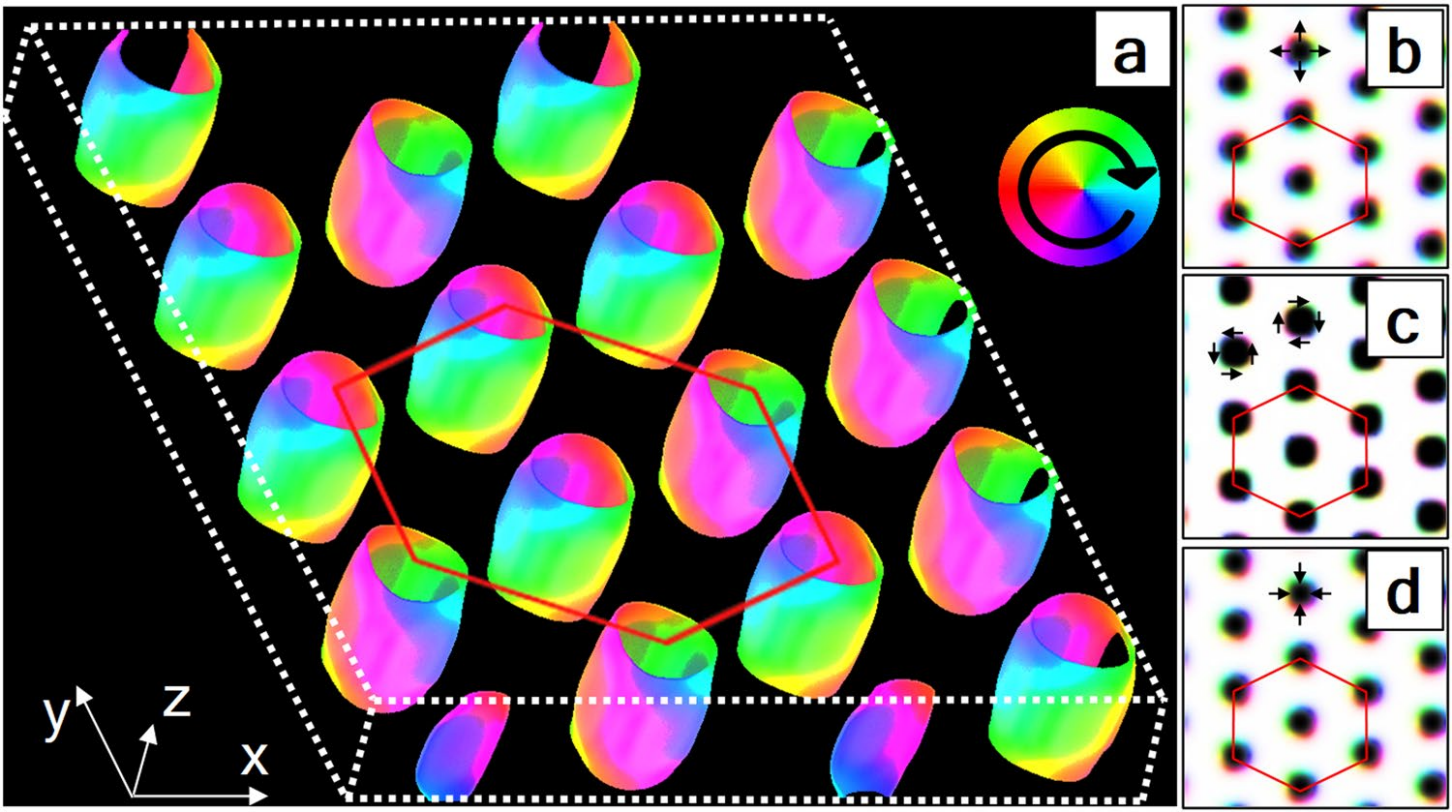
A simulated magnetic bubble domain ground state formed on a quasi-two-dimensional ferromagnetic system. (**a**) A 3D image of the spin configuration with colored in-plane and transparent out-of-plane spin components. Images of (**b**) the top layer (L_*z*_ = 15), (**c**) the middle layer (L_*z*_ = 8), and the bottom layer (L_*z*_ = 0) spin configurations. The red hexagon indicates the hexagonal lattice of the magnetic bubble domain.

We performed a simulation on a 100 × 100 × 15 system (*m* = 15 × 10^5^) by a *k*-space-based algorithm using the Hamiltonian in Eq. (2) with *n* = 8, *J* = 1, $|{\overrightarrow{DM}}_{ij}|=0.0$, ${K}_{{\rm{u}}}={K}_{z}=1.05$, $|{\overrightarrow{h}}_{{\rm{ext}}}|=0.5$, and *D*_dip_ = 0.15. We used periodic boundary conditions in the *x* and *y* directions and non-periodic boundary conditions in the out-of-plane (*z*) direction. Figure 4 shows that our algorithm can generate a bubble domain with a hexagonal lattice structure with Neel cap regions on both surfaces of the quasi-two-dimensional system. Because this bubble domain is formed by dipolar interaction, domain walls coexist with two chiralities, as seen by the color change of a single bubble from the top surface to the bottom surface in Fig. 4. This result proves that the *k*-space-based algorithm can be an effective simulation technique to obtain the ground state of complicated systems.

## Conclusion

We propose an algorithm that obtains ground state spin configurations through an IFFT of hermitized *k*-space information formed by a generative ML model using a simple CVNN.

The network parameters in the proposed algorithm are trained to generate the *k*-space information of energy minimized spin structures. The training results show perfectly long-range ordered structures close to the known magnetic ground states. This shows that the magnetic spin configurations generated using the *k*-space-based algorithm are much closer to the ground state configuration than the results of the *r*-space-based algorithm known as the prior state-of-the-art algorithm.

Additionally, we found that our algorithm can generate well-ordered structures without dependence on the simulation size. Considering that it is very difficult to obtain a long-range ordered ground state result for large system sizes through conventional methods, our algorithm is a very accurate method for obtaining ground states.

Finally, this *k*-space-based algorithm is expected to not only be limited to magnetic systems but also be globally applicable to study other intricately interacting physical systems.
